# The pro‐inflammatory signature of lipopolysaccharide in spontaneous contracting embryoid bodies differentiated from mouse embryonic stem cells

**DOI:** 10.1111/jcmm.17805

**Published:** 2023-06-14

**Authors:** Jennifer Scharmacher, Maria Wartenberg, Heinrich Sauer

**Affiliations:** ^1^ Department of Physiology Justus Liebig University Giessen Giessen Germany; ^2^ Department of Internal Medicine I, Division of Cardiology University Hospital Jena, Friedrich Schiller University Jena Germany

**Keywords:** cardiomyocyte, embryonic stem cells, infection, inflammation, lipopolysaccharide

## Abstract

Embryonic stem (ES) cells differentiate towards all three germ layers, including cardiac cells and leukocytes, and may be therefore suitable to model inflammatory reactions in vitro. In the present study, embryoid bodies differentiated from mouse ES cells were treated with increasing doses of lipopolysaccharide (LPS) to mimic infection with gram‐negative bacteria. LPS treatment dose‐dependent increased contraction frequency of cardiac cell areas and calcium spikes and increased protein expression of α‐actinin. LPS treatment increased the expression of the macrophage marker CD68 and CD69, which is upregulated after activation on T cells, B cells and NK cells. LPS dose‐dependent increased protein expression of toll‐like receptor 4 (TLR4). Moreover, upregulation of NLR family pyrin domain containing 3 (NLRP3), IL‐1ß and cleaved caspase 1 was observed, indicating activation of inflammasome. In parallel, generation of reactive oxygen species (ROS), nitric oxide (NO), and expression of NOX1, NOX2, NOX4 and eNOS occurred. ROS generation, NOX2 expression and NO generation were downregulated by the TLR4 receptor antagonist TAK‐242 which abolished the LPS‐induced positive chronotropic effect of LPS. In conclusion, our data demonstrate that LPS induced a pro‐inflammatory cellular immune response in tissues derived from ES cells, recommending the in vitro model of embryoid bodies for inflammation research.

## 1 INTRODUCTION

Infection, inflammation and subsequent sepsis are major threats of human health and significantly contribute to mortality even in developed countries. In humans, infections with Gram‐negative bacteria, including *Escherichia coli* and *Pseudomonas aeruginosa*, are the prevailing cause of severe sepsis.^1^ Clinical strategies to treat infection and inflammation are principally investigated in animal experiments, for example the caecal ligation and puncture (CLP) model of sepsis, the colon ascendens stent peritonitis model (CASP), the toxaemia model or the murine cutaneous abscess model.^2^ Although a variety of different species is used in infection research, most experiments are performed in mice due to low cost, ease of housing, and their rapid reproduction times and large litter sizes.^3^ In mice, purified LPS (‘endotoxin’) is injected via either the intraperitoneal (i.p.) route or the intravenous (i.v.) route to investigate signalling pathways involved in infection and inflammation.^4^ Due to the ethical questionability of animal experiments and increased opposition of societies against animal experimentation, cellular models of infection were established and are profiting from technological developments in high‐resolution fluorescence microscopy, including long‐term single‐cell time‐lapse microscopy.^5^ However, single cell and monolayer cell models are far away from the complexity of the infection process in organisms, which involves direct effects on different tissues and organs as well as reactions of the innate and acquired immune system. A new experimental approach is multicellular (organoid) cultures consisting of different cell types.^6^ A further promising multicellular 3‐dimensional culture system suitable for in vitro testing is the multicellular embryoid body model grown from ES cells. Within embryoid bodies, stem cells can differentiate towards cells of all three germ layers, including cells of the cardiovascular system and the haematopoietic cell lineage. It has been shown by us and others that embryoid bodies are reactive towards bacterial and viral infection,^7^, ^8^ indicating that they possess essential components of the innate immune system. Previous studies demonstrated that exposure of embryoid bodies derived from mouse ES cells towards LPS resulted in upregulation of the cell adhesion molecules ICAM‐1 and VCAM, which is indicative for the onset of inflammation processes and indicated an early ontogeny for the endothelial cell signal transduction pathway necessary for leukocyte recruitment.^9^ In cardiomyocytes derived from human induced pluripotent stem (iPS), cells LPS increased expression levels of pro‐inflammatory and chemotactic cytokines and induced dysfunction of electrical activity.^10^ LPS is the major component of the outer membrane of Gram‐negative bacteria and is mainly acting via toll‐like receptor 4 (TLR4), which is expressed in cells of the innate immune system, but also in other cell types including cardiac cells, smooth muscle cells^11^ and the endothelium.^12^ In the heart, TLR4 is an essential mediator of inflammatory and apoptosis processes and plays a pivotal role in the development of cardiovascular diseases.^13^ Activation of TLR4 is initiated by binding of the serum lipopolysaccharide (LPS)‐binding protein (LBP) to LPS to facilitate subsequent extraction of LPS monomers by CD14 and the delivery of LPS to the TLR4/MD‐2 complex. TLR4 triggers two signalling pathways called the MyD88‐dependent and the TRIF‐dependent one after the adaptor proteins involved in their induction.^14^ TLR4 is tied to the canonical activation of the NRLP3 inflammasome, which is a multimeric complex of NLRP3, ASC, NEK7 and pro‐caspase 1, and promotes autoproteolysis and activation of caspase 1. The latter cleaves pro‐IL‐1β and pro‐IL‐18 to their secreted pro‐inflammatory forms. Moreover, LPS can directly bind and activate caspase 11, which next stimulates the primed NLRP3 inflammasome and induces IL‐1ß release and pyroptosis.^14^

In the present study, the multicellular EB system derived from murine ES cells was used to investigate LPS‐induced pro‐inflammatory TLR‐4‐mediated signalling pathways. Our data show that LPS treatment of differentiated embryoid bodies exerted tachycardia in beating clusters of cardiac cells, raised ROS and NO, induced differentiation/proliferation of CD68^+^ and CD69^+^ cells, and activated the NLRP3 inflammasome. Thus, the embryoid model is a helpful tool to investigate processes of infection and inflammation in a disease‐in‐dish in vitro setting.

## 2 MATERIALS AND METHODS

### 2.1 Materials

Lipopolysaccharide from *E. coli* O111:B4 was purchased from Sigma‐Aldrich, and TAK‐242 was purchased from Cayman Chemical. VAS2870 was a generous gift from Vasopharm.

### 2.2 Methods

#### 2.2.1 Spinner‐culture technique for cultivation of embryoid bodies

To obtain three‐dimensional embryoid bodies, ES cells (line CCE) were grown on mitotically inactivated feeder layers of primary murine embryonic fibroblasts in Iscove's medium (Gibco, Thermo Fisher Scientific) supplemented with 15% heat‐inactivated (56°C, 30 min) foetal calf serum (FCS) (Sigma‐Aldrich), 2 mM glutamine, (PAA), 100 μM 2‐mercaptoethanol (Sigma‐Aldrich), 1% (v/v) NEA non‐essential amino acids stock solution (100×) (Gibco), 1% (v/v) MEM amino acids (50×) (Gibco), 1 mM Na^+^‐pyruvate (Gibco), 0.4% penicillin/streptomycin (100×) (Gibco), 2.5 μg/mL plasmocin (InvivoGen) and 1000 U/mL LIF (Chemicon) in a humidified environment containing 5% CO_2_ at 37°C, and passaged every 2–3 days. At Day 0 of differentiation, adherent cells were enzymatically dissociated using 0.05% trypsin–EDTA in phosphate‐buffered saline (PBS) (Gibco) and seeded at a density of 3 × 10^6^ cells in 250 mL siliconized spinner flasks (CellSpin, Integra Biosciences) containing 125 mL Iscove's medium supplemented as described above, but devoid of LIF and plasmocin. Following 24 h, 125 mL medium was added to give a final volume of 250 mL. The spinner flask medium was stirred at 20 r.p.m. using a stirrer system (Integra Biosciences). A volume of 125 mL cell culture medium was exchanged on a daily basis. For evaluating contractile activity, approximately 30 embryoid bodies in each individual experiment were plated into 6 cm cell culture dishes. Contractile activity was evaluated by transmission light microscopic inspection at 50‐fold magnification. Maximum contractile activity of embryoid bodies was observed within 10 days of cell culture.

#### 2.2.2 LPS and inhibitor treatment protocol

Embryoid bodies were removed from spinner flasks on Day 3 of differentiation and plated either onto 6 cm Petri dishes or gelatin‐coated glass cover slips in 24‐wells, and further cultivated until Day 9 of cell culture, that is after completion of cardiac cell differentiation. Incubation with 0.1, 1 and 5 μg/mL LPS was performed from Day 9 to Day 15 of cell culture. Thereafter, embryoid bodies were fixed and used for immunohistochemistry experiments. For ROS and NO measurements, embyoid bodies were cultivated in suspension culture until Day 9 of cell culture and incubated with LPS for 48 h. Subsequently, they were stained with ROS‐ and NO‐specific fluorescence dyes and investigated by laser scanning microscopy. In experiments with inhibitors (i.e. VAS2870, TAK‐242), the agents were added to the cell culture medium 24 h before treatment with LPS (i.e. Day 8 of cell culture) and were continuously present in the cell culture medium.

#### 2.2.3 Measurement of ROS generation

Intracellular ROS levels were measured using the fluorescent dye 2′7′‐dichlorodihydrofluorescein diacetate (H_2_DCF‐DA) (Life Technologies, Thermo Fisher Scientific), which is a nonpolar compound that is converted into a nonfluorescent polar derivative (H_2_DCF) by cellular esterases after incorporation into cells. H_2_DCF is membrane impermeable and is rapidly oxidized to the highly fluorescent 2′,7′‐dichlorofluorescein (DCF) in the presence of intracellular ROS. For the experiments, embryoid bodies were incubated in serum‐free medium, and 20 μM H_2_DCF‐DA dissolved in dimethyl sulfoxide (DMSO) was added. After 30 min cells were washed in serum‐free medium, incubated for further 10 min, and intracellular DCF fluorescence (corrected for background fluorescence) was evaluated in 3600 μm^2^ regions of interest using an overlay mask. For the assessment of mitochondrial ROS, embryoid bodies were enzymatically dissociated on Day 7 of cell culture, plated onto cover slips and after 48 h stained for 30 min with the fluorescence dye MitoSox Red (8 μM) (Life Technologies, Thermo Fisher Scientific). For fluorescence excitation of DCF, the 488 nm band of the argon ion laser of a confocal laser scanning microscope (Leica SP2 AOBS, Leica) was used. MitoSox Red was excited at 496 nm. Emission was recorded at an emission band of 515–550 nm for DCF fluorescence and >560 nm for MitoSox Red.

#### 2.2.4 Measurement of NO generation

Nitric oxide generation was evaluated by the use of the cell permeable specific fluorescent NO indicator DAF‐FM diacetate (4‐amino‐5‐methylamino‐2′,7′‐difluorofluorescein diacetate) (Life Technologies, Thermo Fisher Scientific). After incorporation into cells DAF‐FM reacts rapidly with NO to yield a highly fluorescent benzotriazole. Embryoid bodies in suspension culture were incubated for 30 min with 2 μM DAF‐FM diacetate dissolved in cell culture medium. Subsequently, embryoid bodies were washed with serum‐free medium, and cells were incubated for further 20 min on a cell shaker. They were finally transferred to an incubation chamber mounted to the inspection table of the confocal setup, and DAF‐FM fluorescence was recorded in single embryoid bodies (10 embryoid bodies per experiment) using the 488‐nm of the argon‐ion laser of the confocal setup. Emission was recorded at >515 nm. DAF fluorescence was determined using the image analysis software of the confocal setup.

#### 2.2.5 Measurement of intracellular calcium activity in cardiac cells

Embryoid bodies were enzymatically dissociated using trypsin/EGTA solution on Day 9 and plated onto gelatine‐coated coverslips. One day thereafter they were loaded for 10 min in cell culture medium supplemented with 5 μM Fluo‐4,AM. After removal of the staining medium and supplementation with fresh cell culture medium, coverslips were transferred to an incubation chamber, which was mounted to the stage of the confocal microscope. Excitation was performed using the 488 nm line of an argon ion laser. Emission was recorded at 515–525 nm. Fluo 4 fluorescence is displayed as F/F_0_, where F is the actual Fluo 4 fluorescence devided by the basal fluorescence level F_0_.

#### 2.2.6 Immunohistochemistry and confocal imaging

As primary antibodies mouse monoclonal anti α‐actinin antibody (Sigma‐Aldrich, cat. no. A7811), rabbit polyclonal anti cardiac troponin T (cTnT) (Abcam, cat. no. ab209813), rat monoclonal anti NLRP3 (Invitrogen, Thermo Fisher Scientific, cat. no. MA5–25318), rabbit polyclonal anti CD68 (Bioss, cat. no. Bs – 0649R), rabbit polyclonal anti TLR4 (Invitrogen, Thermo Fisher Scientific, cat. no. 710185), rabbit polyclonal anti IL‐1ß (Invitrogen, Thermo Fisher Scientific, cat. no. PA5‐88078) and rabbit polyclonal CD69 (Invitrogen, Thermo Fisher Scientific, cat. no. PA5‐114989) were used. Either whole mount embryoid bodies outgrown on cover slips or single cells dissociated from embryoid bodies and cultivated on coverslips were fixed in 4% paraformaldehyde on ice for 20 min, washed 1–2 times with phosphate‐buffered saline (PBS) supplemented with 0.01% Triton‐X‐100 (Sigma‐Aldrich) (0.01% PBST) and permeabilized for 10 min with 1% PBST. Blocking against unspecific binding was performed for 60 min with 10% fat‐free milk powder dissolved in 0.01% PBST (blocking solution). The cells were subsequently incubated overnight with primary antibody dissolved in blocking solution. Cells were thereafter washed three times with PBST (0.01% Triton) and re‐incubated for 1 h at room temperature in dark with either a Cy3 goat anti‐rabbit, Cy3 goat anti mouse or an Alexa 488 donkey anti‐rat IgG (Molecular Probes, Thermo Fisher Scientific) (dilution 1:100) in blocking solution. After washing three times in 0.01% PBST, the cells were stored in PBS until inspection. For staining of cell nuclei, the nuclear stain DRAQ5 (Thermo Fisher Scientific) was used (dilution 1:2000) and excited at 633 nm. Fluorescence recordings were performed by means of a confocal laser scanning setup (Leica TCS SP2). The confocal setup was equipped with a 5 mW helium/neon laser, single excitation 633 nm, a 0.5 mW helium/neon laser, single excitation 543 nm, and an argon ion laser, single excitation 488 nm.

#### 2.2.7 Flow cytometry

Embroid bodies in suspension culture were treated from Day 9 to Day 15 of cell culture with LPS (5 μg/mL). Subsequently, they were dissociated using collagenase B (Sigma‐Aldrich), pelleted, fixed in paraformaldehyde (4%) on ice for 20 min and permeabilized in 0.1% PBST. Blocking against unspecific binding was performed in 10% BSA for 1 h. Cells were resuspended in FACS buffer [0.1% sodium azide, 5% foetal calf serum (FCS), 0.05% in PBS], and stained with either rabbit polyclonal anti CD68 (Bioss, cat. no. Bs – 0649R) or rabbit polyclonal CD69 (Invitrogen, Thermo Fisher Scientific, cat. no. PA5‐114989) antibodies. FITC‐conjugated anti‐rabbit antibodies were used as secondary antibodies. Flow cytometry data acquisition was carried out by FACSCalibur cytometer (BD Biosciences) and BD CellQuest Pro software (BD). Data are represented as the percentage of positive‐stained cells per total counted cells in each experiment.

#### 2.2.8 Western blot analysis

The western blot assays were carried out after washing embryoid bodies in PBS and lysing in RIPA lysis buffer [50 mM Tris–HCl (pH 7.5), 150 mM NaCl, 1 mM EDTA (pH 8.0), 1 mM glycerophosphate, 0.1% SDS, 1% Nonidet P‐40] supplemented with protease inhibitor cocktail (Biovision) and phosphatase inhibitor cocktail (Sigma‐Aldrich) for 20 min on ice. Samples were centrifuged at 13,000*g* for 10 min at 4°C to pellet the debris. After determination of the protein concentration using a Lowry protein assay, 30 μg of protein samples was heated for 10 min at 70°C, separated in NUPAGE 4–12% Bis‐Tris gradient mini gels and transferred to nitrocellulose membranes by the XCell SureLock Mini‐Cell Blot Module (Invitrogen) at 150 V for 90 min. Membranes were either blocked with 5% (wt/vol) dry fat‐free milk powder or 5% BSA in Tris‐buffered saline with 0.1% Tween (TBST) for 60 min at room temperature. Incubation with primary antibody was performed at 4°C overnight. Used primary antibodies were as follows: mouse anti α‐actinin (dilution 1:1000) (Sigma‐Aldrich, cat. no. A5044), rabbit anti cleaved caspase 1 (dilution 1:1000) (Thermo Fisher Scientific, cat. no. PA5–38099), rabbit anti cleaved caspase 3 (dilution 1:1000) (Cell Signaling, cat. no. D175 5AIE), rabbit anti CD68 (dilution 1:1000) (Bioss, cat. no. bs‐0649R), rabbit anti‐CD69 (dilution 1:1000) (Thermo Fisher Scientific, cat. no. PA5‐114989), rabbit anti Enos (dilution 1:1000) (Sigma‐Aldrich, cat. no. N13893), rabbit anti NOX1 (dilution 1:3000) (Novus Biologicals, cat. no. NBP1‐31546), rabbit anti NOX2 (dilution 1:10,000) (Proteintech, cat. no.19013‐1‐AP), rabbit anti NOX4 (dilution 1:1000) (Abcam, cat. no. NBP1‐31546), rabbit anti pEnos (dilution 1:1000) (Abcam, cat. no. ab215717), mouse anti TLR 4 (dilution 1:1000) (Thermo Fisher, cat. no. MA5‐16216) and rabbit anti vinculin (dilution 1:1000) (Abcam, cat. no. ab129002). After washing three times with 0.1% TBST for at least 10 min, the membrane was incubated with a horseradish peroxidase (HRP)‐conjugated secondary antibody (dilution 1:1000) (Abcam, cat. no. ab205722, ab205724) for 60 min at room temperature. After the washing process with 0.1% TBST, the blot was developed using ECL to produce a chemiluminescence signal. For quantification, the density of protein bands on the western blot image, which was acquired using the peqlab gel documentation system (VWR), was assessed by Image J. The final quantification reflects the relative amounts of protein as a ratio of each target protein band to the respective housekeeping protein.

### 2.3 Statistical analysis

Data are given as mean values ± SD, with *n* denoting the number of experiments unless otherwise indicated. One‐way anova for unpaired data and Student's *t*‐test were applied as appropriate. A value of *p* ≤ 0.05 was considered significant.

## 3 RESULTS

### 3.1 Expression of TLR4 in embryoid bodies upon LPS treatment

**FIGURE 1:**
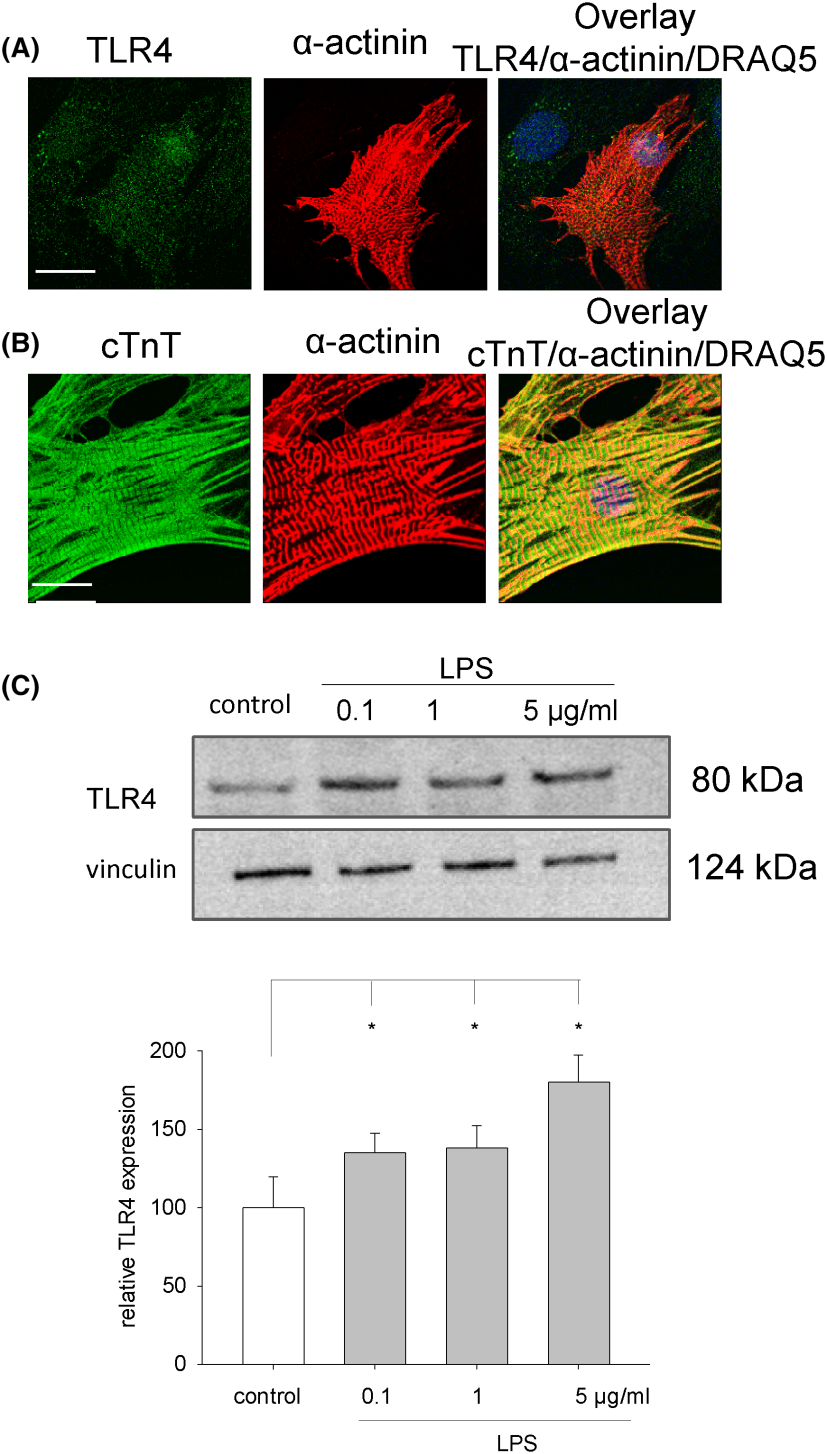
Expression of TLR4 in cardiac cells differentiated from ES cells and embryoid bodies. (A) Immunohistochemistry of TLR4 expression in single cardiac (α‐actinin‐positive) and non‐cardiac (α‐actinin‐negative) cells enzymatically isolated from 15‐day‐old embryoid bodies. Left image, TLR4 (green); middle image, α‐actinin (red); right image, overlay DRAQ5 nuclear staining (blue), TLR4 (green), α‐actinin (red) (*n* = 3). The scale bar represents 25 μm. (B) Expression of cTnT (green) and α‐actinin (red) in cardiomyocytes. The cell nuclei (blue) were labelled with DRAQ5 and are shown in the overlay image. (C) Western blot analysis of TLR4 expression in 15‐day‐old embryoid bodies treated with 0.1, 1 and 5 μg/mL LPS. Upper image, representative western blot, lower image, bar chart showing the means ± SD of *n* = 3 experiments. *p < 0.05, significantly different as compared to the untreated control.

It is generally accepted that the inflammatory response of LPS is mediated via TLR4.^15^ We therefore investigated, whether incubation with LPS would upregulate TLR4, and whether TLR4 is expressed in cardiomyocytes differentiated from ES cells. The data of the present study demonstrate that TLR4 was expressed in 10‐day‐old single cardiac and non‐cardiac cells enzymatically dissociated from embryoid bodies (Figure 1A). Cardiac cells were identified by double‐labelling with antibodies against sarcomeric α‐actinin and cTnT (Figure 1B). Moreover, TLR4 protein in embryoid bodies was dose‐dependent upregulated by LPS upon treatment from Day 9 to Day 15 of cell culture (Figure 1C).

### 3.2 Contractile activity of embryoid bodies upon incubation with LPS

**FIGURE 2:**
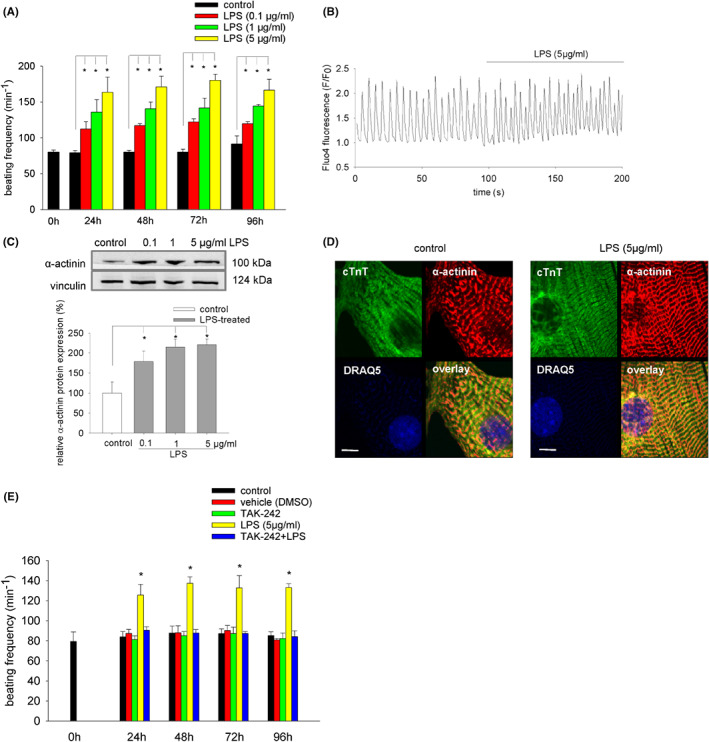
Effect of LPS on beating frequency of embryoid bodies, calcium spikes and α‐actinin/cTnT expression in cardiac cells. (A) Analysis of beating frequency (min^−1^) in plated embryoid bodies, which were treated from Day 9 of cell culture for a maximum of 96 h with either 0.1, 1 or 5 μg/mL LPS. Beating frequency was analysed every 24 h by transmission light microscopical inspection at 50‐fold magnification (*n* = 3). (B) Calcium transients in single cardiac cells enzymatically isolated on Day 9 from beating embryoid bodies and treated 24 h thereafter with LPS (5 μg/mL) (*n* = 4). The cells attached on coverslips were loaded with the calcium indicator Fluo‐4,AM. Cardiac cells were identified by rhythmic Fluo‐4 fluorescence changes. Shown is the change in fluorescence intensity relative to baseline (*F*/*F*_*0*_). (C) α‐actinin expression in embryoid bodies, which were treated from Day 9 to Day 15 with LPS. Upper panel, representative western blot, lower panel, bar chart of the means ± SD of *n* = 3 experiments. (D) α‐actinin (red), cTnT (green) immunohistochemistry and nuclear staining with DRAQ5 (blue) in single cardiac cells enzymatically dissociated from embryoid bodies treated from Day 9 to Day 15 with 5 μg/mL LPS (*n* = 3). The scale bar represents 6 μm. *p < 0.05, significantly different as compared to the untreated control. (E) Effect of the TLR4 inhibitor TAK‐242 (1 μM) on the beating frequency of embryoid bodies treated from Day 9 of cell culture for a maximum of 96 h with 5 μg/mL LPS either in presence or absence of TAK‐242 (added 24 h before treatment with LPS). Beating activity was analysed by microscopical inspection every 24 h (*n* = 3). *p < 0.05, significantly different as compared to the untreated control.

Within 10 days of cell culture embryoid bodies differentiate cardiac areas and develop contractile activity. To investigate the effect of LPS treatment on beating frequency, 9‐day‐old embryoid bodies outgrown on coverslips were treated with increasing concentrations of LPS (0.1, 1, 5 μg/mL) and contraction frequency was analysed every 24 h. It was evident (Figure 2A) that beating frequency was significantly and dose‐dependent increased as early as 24 h following incubation with LPS, indicating emergence of tachycardia. Moreover, the frequency of intracellular calcium transients occurring during contractions was increased upon addition of LPS (5 μg/mL) to the incubation medium (Figure 2B), as evaluated by fluo‐4 microfluorometry. Possible structural changes in cardiomyocytes upon LPS treatment were assessed by western blot analysis of α‐actinin expression (Figure 2C) as well as immunohistochemistry of the sarcomere structure of α‐actinin and cTnT‐positive cardiac cells (Figure 2D). Our data evidenced that LPS treatment dose‐dependent increased α‐actinin protein expression. However, the sarcomere structure of cardiomyocytes remained unchanged (Figure 2D). The stimulation of beating frequency by LPS (5 μg/mL) was totally abolished in presence of the TLR4 inhibitor TAK‐242 (1 μM), indicating involvement of TLR4 signalling in the observed effect (Figure 2E). To assess whether the applied concentration range of LPS (0.1–5 μg/mL) was non‐toxic, cytotoxicity was evaluated by analysis of the number cell nuclei positive for the lethal cell marker Sytox green. It was observed that in the applied concentration range of LPS and experimental conditions, that is incubation with LPS from Day 9 to Day 15 of cell culture, no overt toxicity occurred (data not shown).

### 3.3 Differentiation of leukocytes in embryoid bodies following treatment with LPS

**FIGURE 3:**
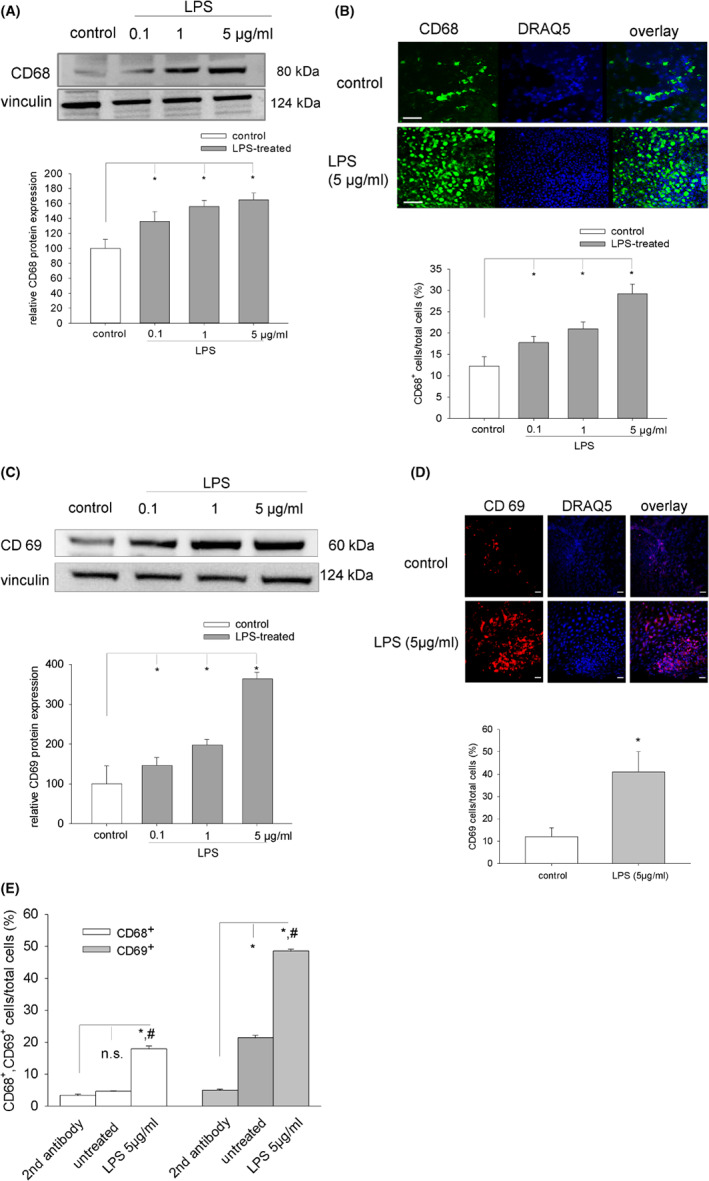
Expression of CD68 and CD69 in embryoid bodies treated with LPS. (A) Western blot analysis of CD68 expression in embryoid bodies treated from Day 9 to Day 15 of cell culture with 0.1, 1 and 5 μg/mL LPS. Upper panel, representative western blot, lower panel, bar chart of the means ± SD of *n* = 3 experiments. (B) Immunohistochemical analysis of CD 68 expression in plated embryoid bodies treated with different concentrations of LPS. Upper panel, representative images of CD68^+^ cells (left, green), DRAQ5 nuclear staining (middle, blue) and overlay (right). Lower panel, bar chart of the means ± *n* = 3 experiments. (C) Western blot analysis CD69 expression in embryoid bodies treated from Day 9 to Day 15 of cell culture with 0.1, 1 and 5 μg/mL LPS. Upper panel, representative western blot, lower panel, bar chart of the means ± SD of *n* = 3 experiments. (D) Immunohistochemical analysis of CD 69 expression in plated embryoid bodies treated with different concentrations of LPS. Upper panel, representative images of CD69^+^ cells (left, green), DRAQ5 nuclear staining (middle, blue) and overlay (right). Lower panel, bar chart of the means ± *n* = 3 experiments. The scale bars in immunohistochemical images represent 30 μm. (E) Flow cytometry analysis of CD68^+^ and CD69^+^ cells/total cells (%) (*n* = 3). Cells were enzymatically dissociated from embryoid bodies treated from Day 9 to Day 15 of cell culture with 5 μg/mL LPS. *p < 0.05, significantly different as compared to 2nd antibody, ^#^ *p* < 0.05, significantly different as compared to the untreated control.

The induction of inflammation in embryoid bodies may be associated with a stimulation of leukocyte differentiation, which has previously shown by us to occur within 15 days of cell culture.^16^ In the present study, protein expression of the macrophage marker CD68 and CD69, which is expressed upon activation of T lymphocytes and natural killer (NK) cells, was investigated. Moreover, the number of CD68^+^ and CD69^+^ cells was determined by confocal microscopy and flow cytometry upon treatment of embryoid bodies with LPS (5 μg/mL). Our data demonstrated that LPS treatment from Day 9 to Day 15 of embryoid body cell culture dose‐dependent increased protein expression of CD68 (Figure 3A) and CD69 (Figure 3C). Moreover, upon incubation with LPS the number of CD68^+^ and CD69^+^ cells increased from 12% in the untreated control to 29% and 41%, respectively, in the treated sample (Figure 3B,D), indicating differentiation and/or proliferation of leukocytes under these experimental conditions. These data were confirmed by flow cytometry analysis of CD68^+^ and CD69^+^ cells enzymatically dissociated from embryoid bodies (Figure 3E).

### 3.4 Generation of ROS and expression of NOX enzymes upon LPS treatment

**FIGURE 4:**
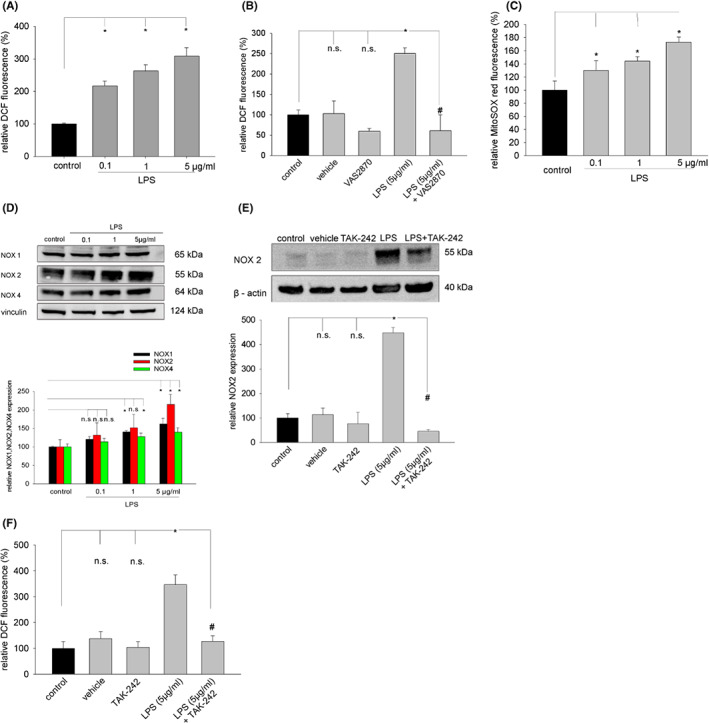
ROS generation and expression of NOX enzymes upon treatment of embryoid bodies with LPS. Embryoid bodies in suspension culture were treated from Day 9 to Day 11 of cell culture with 0.1, 1 and 5 μg/mL LPS, respectively, and labelled with the ROS‐sensitive fluorescence dye H_2_DCF‐DA (A, B). ROS generation in mitochondria was assessed in single cells enzymatically dissociated from embryoid bodies using the mitochondria‐specific ROS indicator MitoSOX red (C). DCF and MitoSOX red fluorescence was monitored by laser scanning microscopy. (A) Dose‐dependent increase in cytoplasmic ROS generation upon treatment with LPS (*n* = 3). (B) Inhibition of LPS (5 μg/mL)—induced ROS by the NADPH oxidase inhibitor VAS2870 (20 μM) (*n* = 3). VAS2870 was added 24 h before treatment with LPS and was continuously in the cell culture medium. (C) Dose‐dependent increase in mitochondrial ROS generation upon treatment with LPS (*n* = 3). (D) Western blot of NOX1, NOX2 and NOX4 protein expression upon treatment of embryoid bodies from Day 9 to Day 15 with different concentrations of LPS. Upper panel, representative western blot, vinculin was used as loading control. Lower panel, bar chart of the means ± SD of *n* = 3 experiments. Shown is the relative NOX1, NOX2, and NOX4 expression. (E) Effect of the TLR4 inhibitor TAK‐242 on NOX2 protein expression. Upper panel, representative western blot of embryoid bodies treated from Day 9 to Day 15 with vehicle (DMSO), TAK‐242 (1 μM), LPS (5 μg/mL) or a combination of LPS and TAK‐242. ß‐actin was used as loading control. Lower panel, bar chart of the means ± SD of *n* = 3 experiments. Shown is the relative NOX2 protein expression. (F) Inhibition of LPS (5 μg/mL) – induced ROS generation by the TLR4 inhibitor TAK‐242 (1 μM) (*n* = 3). *p < 0.05, significantly different as compared to the untreated control. ^#^ *p* < 0.05, significantly different to the LPS‐treated sample.

Inflammatory processes are associated with oxidative stress arising from ROS, which may generate by NOX enzymes and/or as side products of the mitochondrial respiratory chain. By use of the cytoplasmic ROS indicator H_2_DCF‐DA it was shown, that LPS treatment dose‐dependent increased ROS generation (Figure 4A), which was totally abolished in presence of the NADPH oxidase inhibitor VAS2870 (20 μM) (Figure 4B). Moreover, the mitochondrial ROS indicator MitoSOX red displayed increased mitochondrial fluorescence upon LPS treatment (Figure 4C), indicating that ROS were at least partially generated within the respiratory chain. A possible participation of NOX enzymes was analysed in western blot experiments using antibodies against NOX1, NOX2 and NOX4. LPS treatment dose‐dependent increased the expression of NOX enzymes (Figure 4D). Most prominent changes in protein expression were observed for NOX2. These data indicate involvement of NADPH oxidases in the oxidative stress elicited by LPS in embryoid bodies. Notably, treatment of embryoid bodies with TAK‐242 (1 μM) significantly downregulated the LPS‐induced stimulation of NOX2 expression (Figure 4E) as well as the generation of ROS (Figure 4F), indicating participation of ROS in the TLR4 signalling cascade.

### 3.5 Generation of NO upon treatment of embryoid bodies with LPS

**FIGURE 5:**
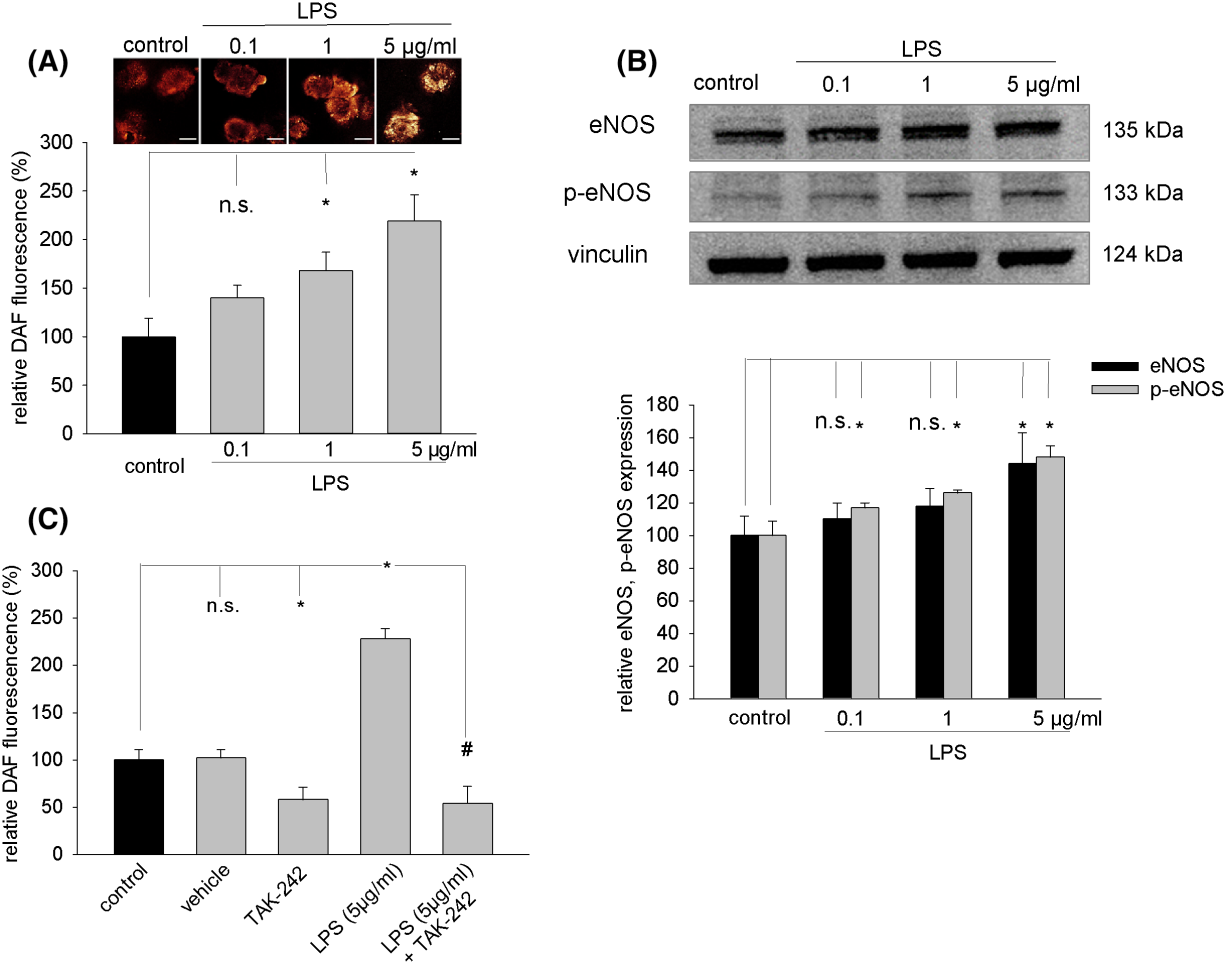
NO generation and expression of eNOS upon treatment of embryoid bodies with LPS. Embryoid bodies in suspension culture were treated from Day 9 to Day 11 of cell culture with 0.1, 1 and 5 μg/mL LPS, respectively, and labelled with the NO‐sensitive fluorescence dye DAF‐FM. (A) Dose‐dependent increase in cytoplasmic NO generation upon treatment with LPS (*n* = 3). The upper panel shows representative DAF‐labelled embryoid bodies in suspension culture. DAF fluorescence was assessed in 10 embryoid bodies in each experiment and analysed using the image analysis software of the confocal setup. The scale bars represent 300 μm. (B) Western blot of relative eNOS and phosphor eNOS protein expression upon treatment of embryoid bodies from Day 9 to Day 15 with different concentrations of LPS. Upper panel, representative western blot, vinculin was used as loading control. Lower panel, bar chart showing the means ± SD of relative p‐eNOS and eNOS expression in *n* = 3 experiments. (C) Inhibition of LPS (5 μg/mL) – induced NO generation by the TLR4 inhibitor TAK‐242 (1 μM) (*n* = 3). TAK‐242 was added 24 h before treatment with LPS and was continuously in the cell culture medium. *p < 0.05, significantly different as compared to the untreated control. ^#^ *p* < 0.05, significantly different to the LPS‐treated sample.

Lipopolysaccharide has been reported to raise NO in previous studies.^17^ To investigate, whether comparable effects would occur in embryoid bodies, the NO‐specific fluorescence dye DAF‐FM was used in microfluorometric experiments. Indeed, we observed that NO generation increased dose‐dependent upon incubation with LPS (Figure 5A). Moreover, LPS treatment dose‐dependent increased protein expression of NOS3 as well as phospho‐NOS3 (Figure 5B). The increase in NO generation elicited by LPS (5 μg/mL) was completely abolished in presence of TAK‐242 (1 μM) (Figure 5C and Figure S1), indicating activation of TLR4 signalling under these experimental conditions. Interestingly TAK‐242 (1 μM) partially downregulated the intrinsic NO generation of embryoid bodies, which may suggest that inflammatory signalling cascades may take place during early embryogenesis.^18^

### 3.6 Activation of the NLRP3 inflammasome in embryoid bodies and ES cell‐derived cardiac cells by LPS

**FIGURE 6:**
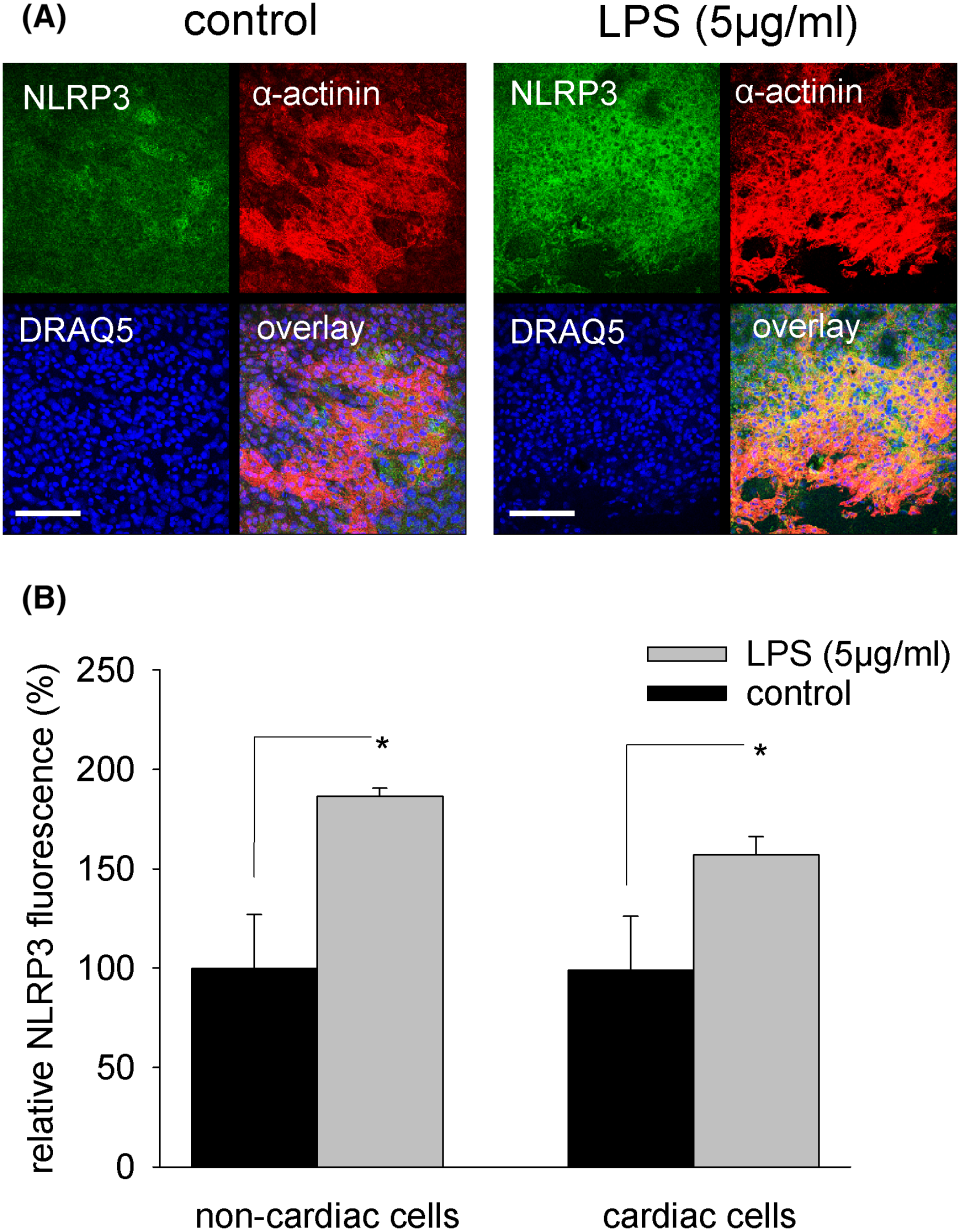
Expression of NLRP3 in cardiac and non‐cardiac cells upon treatment with LPS. Outgrown embryoid bodies were treated from Day 9 to Day 15 with LPS (5 μg/mL), and NLRP3 expression was analysed in α‐actinin‐positive cardiac and non‐cardiac cell areas. (A) Representative images of cardiac areas labelled with an anti‐NLRP3 antibody (green), an anti‐ α‐actinin antibody (red) and the nuclear stain DRAQ5 (blue). Left panel, control, right panel, LPS (5 μg/mL). The scale bars represent 100 μm. (B) Bar chart of semi‐quantitative NLRP3 expression in non‐cardiac and cardiac cell areas as analysed by confocal laser scanning microscopy and computer‐assisted image analysis (*n* = 3). *p < 0.05, significantly different as compared to the untreated control.

**FIGURE 7:**
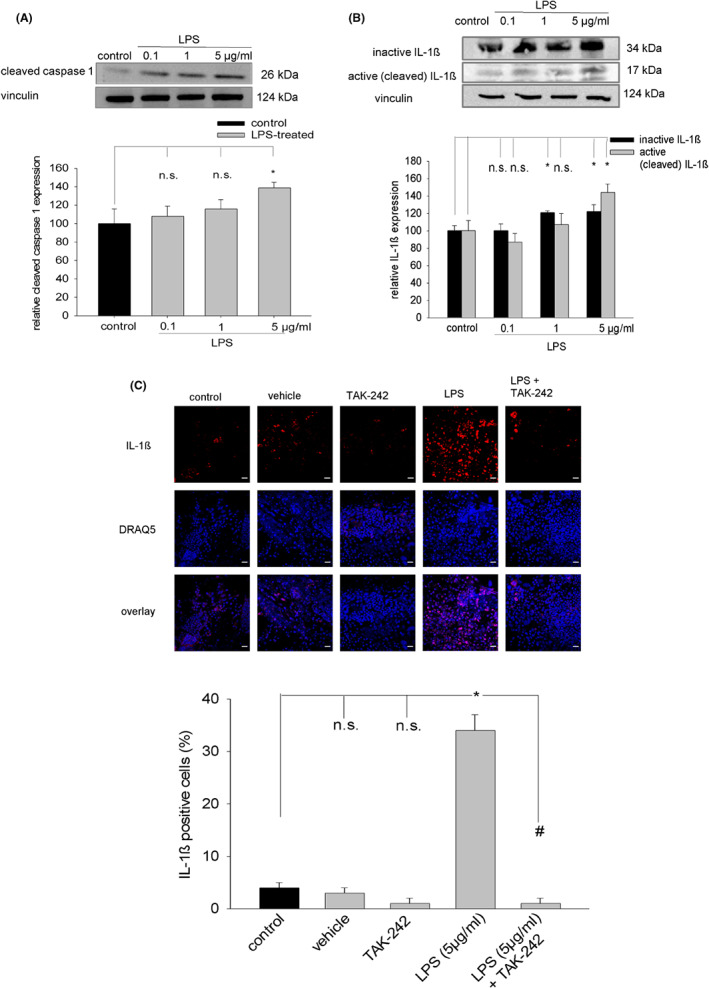
Expression of cleaved caspase 1 and IL‐1ß upon treatment of embryoid bodies with LPS. (A) Western blot analysis of cleaved caspase 1 expression. Upper panel, representative western blot of cleaved caspase 1 upon treatment with either 0.1, 1 or 5 μg/mL LPS from Day 9 to Day 15 of cell culture. Vinculin was used as loading control. Lower panel, bar chart of mean values ± SD of *n* = 3 experiments. (B) Western blot analysis of inactive and active (cleaved) IL‐1ß upon treatment with either 0.1, 1 or 5 μg/mL LPS from Day 9 to Day 15 of cell culture. Vinculin was used as loading control. Upper panel, representative plot. Lower panel, bar chart of mean values ± SD of *n* = 3 experiments. *p < 0.05, significantly different as compared to the untreated control. (C) Effect of the TLR4 inhibitor TAK‐242 (added on Day 8 of cell culture) on the number of IL‐1ß^+^ cells upon incubation of outgrown embryoid bodies for 5 days with vehicle, TAK‐242 (1 μM), LPS (5 μg/mL), and a combination of LPS and TAK‐242. Upper panel, representative images, IL‐1ß (red), DRAQ5 (blue), overlay (blue, red). The scale bars represent 30 μm. Lower panel, bar chart showing the means ± SD of *n* = 3 experiments. *P < 0.05, significantly different as compared to the untreated control. ^#^ *p* < 0.05, significantly different to the LPS‐treated sample.

Lipopolysaccharide and LPS‐induced oxidative/nitrosative stress may induce the NLRP3 inflammasome with concomitant activation of the protease caspase 1. We therefore performed immunohistochemical experiments using an anti‐NLRP3 antibody to investigate whether NLRP3 expression would increase in cardiac versus non‐cardiac cells upon treatment with LPS (5 μg/mL). Our data demonstrated that basal NLRP3 expression was present already in unstimulated non‐cardiac as well as cardiac cells enzymatically dissociated from 15‐day‐old embryoid bodies. However, treatment of embryoid bodies from Day 9 on with LPS (5 μg/mL) significantly upregulated NLRP3 expression in cardiac (Figure 6A,B) as well as non‐cardiac (Figure 6B) cells. Concomitant with NLRP3 protein, expression of active (cleaved) caspase 1 was upregulated in LPS‐treated embryoid bodies (Figure 7A). Moreover, cleaved caspase 3 was non‐significantly induced (data not shown). The assembled NLRP3 inflammasome can facilitate the release of IL‐1β. The data of the present study demonstrated that LPS (5 μg/mL) treatment of embryoid bodies increased expression of inactive as well as active IL‐1ß (Figure 7B). Moreover, LPS significantly increased the number of IL‐1ß‐positive cells, which was completely abolished in presence of the TLR4 inhibitor TAK‐242 (Figure 7C).

## 4 DISCUSSION

Embryoid bodies derived from ES cells are complex, multicellular tissues, which differentiate cells of all three germ layers including cells of the cardiovascular system and immune‐competent cells. They may be therefore suitable to investigate mechanisms of innate immune system activation and the complexity of inflammatory processes, thereby avoiding cost‐intensive and ethically questionable animal experiments. The data of the present study demonstrated that LPS induced tachycardia in spontaneously contracting embryoid bodies. Previous studies performed on isolated cardiomyocytes from human iPS cells evidenced TLR4‐mediated changes in inflammatory gene and cardiac ion channel expression as well as prolongation of action potential duration.^10^ In confirmation with this study, our data demonstrated TLR4 expression in isolated cardiac cells as well as overall upregulation of TLR4 in the multicellular tissue upon LPS treatment. The non‐cardiac tissue, consisting among others of blood‐vessel‐like structures and immune cells is participating in the pro‐inflammatory immune response, may affect heart cell function and modulate LPS effects on the vitality and electrical properties of cardiomyocytes. Increase in heart rate is a known feature of septic shock, and prolonged elevation of the heart rate has been associated with poor outcome.^19^ Tachycardia is arising from stimulation of ß1 adrenergic receptors through stimulation of the sympathetic nerve system. Consequently, sepsis‐mediated tachycardia is clinically treated with ß‐blockers, which significantly decreases the mortality of septic patients.^20^ Epinephrine determines cardiac performance beginning shortly after the first myocardial contractions occur in developing vertebrate embryos.^21^ Previous studies have demonstrated that ES cell‐derived cardiac cells of mouse and human origin are responding towards adrenergic receptor stimulation.^22^, ^23^, ^24^, ^25^ Moreover, recent evidence has suggested that in cardiac tissues intrinsic cardiac adrenergic (ICA) cells exist, which may regulate both developing and adult cardiac physiological and pathological processes and may contribute to LPS‐induced myocardial dysfunction.^26^ Previous studies have shown that the adrenergic biosynthetic enzyme, phenylethanolamine *n*‐methyltransferase (Pnmt) which catalyses the final step in the adrenergic biosynthetic pathway, the conversion of norepinephrine into epinephrine is expressed in the heart of different species during embryogenesis and in the adult. It has been hypothesized that adrenergic cells contribute directly to myocardial cell development through trans‐differentiation from an adrenergic phenotype into a myocyte phenotype.^27^ Since embryoid bodies do not contain an autonomous nerve system, we assumed that LPS is either directly simulating adrenergic receptors or induces the release of catecholamines in the embryoid body tissue. Notably, in the present study the TLR4 inhibitor TAK‐242 abolished the increase in contraction frequency, thus underscoring involvement of TLR4 signalling pathways.

Lipopolysaccharide treatment upregulated protein expression of CD68 and CD69 as well as CD68^+^ and CD69^+^ cell numbers. CD68 is a well‐known marker of macrophages, and CD69 is expressed upon activation of T lymphocytes and natural killer (NK) cells. Differentiation of cells of the innate immune system through specific small molecule‐based protocols from mouse and human ES cells has been previously reported.^28^, ^29^, ^30^ The data of the present study suggest that LPS stimulated either the differentiation of haematopoietic precursors or the proliferation of already preformed leukocytes. Previous studies of us demonstrated that 18‐day‐old embryoid bodies of the ES cell line CGR8 contained immune cells, which were positive for CD45, CD68, CD11b, F4/80 and CD19, and were able to phagocytize bacteria.^7^ In the incubation protocol used in the present study, embryoid bodies were treated from Day 9 to Day 15 of cell culture. Maximum endogenous expression of the pan‐leukocyte marker CD45 was shown to occur on Day 12 of cell culture.^16^ In this time window, cardiovascular differentiation of mouse ES cells is already accomplished. Activation of T lymphocytes, NK cells and macrophages by LPS involves active generation of ROS and NO, which can react towards highly reactive peroxynitrite species. A major player in ROS generation is NOX2, which is expressed in various cell types, such as macrophages, cardiac cells, endothelial cells, dendritic cells, B and T lymphocytes.^31^, ^32^ In the present study, robust upregulation of NOX2 was observed. Consequently, LPS treatment resulted in ROS generation, which was inhibited by the NADPH oxidase antagonist VAS2870. Moreover, using the mitochondrial ROS indicator MitoSOX red, generation of ROS, presumably via the respiratory chain, was evidenced. NOX2 is the major NADPH oxidase isoform involved in the killing of bacteria by macrophages and T‐cell receptor activation of cytotoxic CD8^+^ T‐lymphocytes.^33^ Moreover, NADPH oxidases are necessary for macrophage differentiation.^34^ Besides NOX2, NOX1 and NOX4 were moderately upregulated. Activation of NOX2 by LPS has been previously shown in human umbilical vein endothelial cells.^35^ In mice lacking NOX2, LPS failed to increase ROS generation. NOX1 was shown to be involved in LPS‐induced cardiomyocyte apoptosis by increasing oxidation of Akt and subsequent dephosphorylation by PP2A.^36^ Moreover, it was demonstrated that TLR4 is directly interacting with NOX4.^37^

In parallel to the upregulation of NOX enzymes LPS increased the expression of eNOS and phosphor eNOS, which was accompanied by increased NO generation. Previous studies have demonstrated that injection of LPS into rats increased cardiac levels of the peroxynitrite precursors NO and superoxide which reacted towards peroxynitrite.^38^ Enhanced peroxynitrite formation was previously detected in granulocytes, monocytes, lymphocytes and plasma cells after challenge with LPS.^39^ Notably, the data of the present study indicated that pre‐treatment with TAK‐242 significantly blunted the LPS‐induced expression of NOX2 and inhibited ROS production. Moreover, LPS‐induced NO generation was completely abolished under these experimental conditions. Interestingly TAK‐242 significantly depressed the endogenous NO production in the absence of LPS, which may indicate that TLR4 is involved in basal NO generation by embryoid bodies.

Lipopolysaccharide is eliciting an inflammatory response via the NLRP3 inflammasome. Indeed, the data of the present study demonstrated increased expression of NLRP3 in cardiac and non‐cardiac cells, which suggests a general pro‐inflammatory microenvironment within the tissue. NLRP3 promotes autoproteolysis and activation of caspase 1, which was confirmed in the present study. Moreover, activation of caspase 3 was observed, which remained, however, non‐significant. Since caspase 1 cleaves pro‐IL‐1β protein, the number of IL‐1ß^+^ cells was assessed. Indeed, LPS treatment dose‐dependent increased IL‐1ß protein expression and the number of IL‐1ß^+^ cells. Pre‐treatment with TAK‐242 totally abolished this effect.

Lipopolysaccharide‐induced activation of the NLRP3 inflammasome has been above all investigated in laboratory animals and specific cell types, but so far not in complex tissues consisting of organotypic cells, which interact—comparably to a living organism—in a coordinated manner during the inflammatory process. The data of the present study demonstrate that LPS‐induced inflammation with its complex players can be assessed in ES cell‐derived multicellular tissues. From these tissues, single cells like cardiac cells or cells of the innate immune system can be enzymatically dissociated and inflammation‐associated pathways compared with the multicellular tissue situation. Inflammation, activation of the innate immune system and the underlying signal transduction pathways are a major challenge for future research since sepsis due to bacterial or viral infection is worldwide affecting millions of patients. In light of increasing antibiotic resistance of bacteria, lack of effective treatment for viral infection and immune escape of pathogens in vaccinated people, a powerful high throughput multicellular in vitro test system of the innate immune system is needed to push forward the endeavours for an effective clinical treatment of infection, inflammation and sepsis.

## AUTHOR CONTRIBUTIONS

**Jennifer Scharmacher:** Data curation (equal); investigation (lead). **Maria Wartenberg:** Conceptualization (equal); formal analysis (equal); project administration (equal); writing – review and editing (supporting). **Heinrich Sauer:** Conceptualization (lead); funding acquisition (lead); investigation (equal); supervision (lead); writing – original draft (lead).

## CONFLICT OF INTEREST STATEMENT

The authors confirm that there are no conflicts of interest.

## Supporting information


Fig. S1:

## Data Availability

The data that support the findings of this study are available from the corresponding author upon reasonable request.
