# Supplementary figures and images for: The pro‐inflammatory signature of lipopolysaccharide in spontaneous contracting embryoid bodies differentiated from mouse embryonic stem cells
Source: J Cell Mol Med. 2023 Jun 14;27(14):2045–58. doi: 10.1111/jcmm.17805 (PMC10339090; doi:10.1111/jcmm.17805)

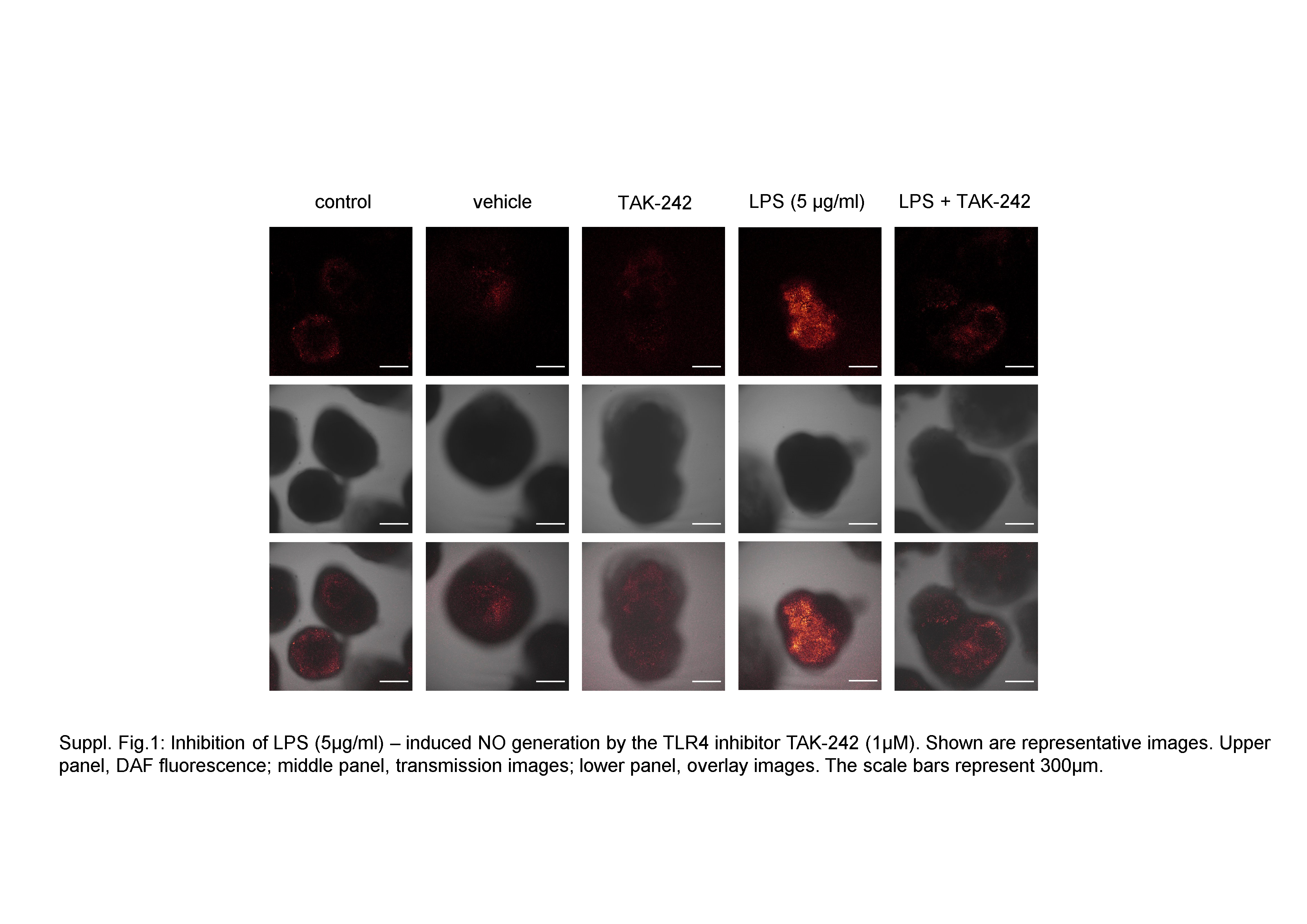

Supplement: Supplementary file 1 — Fig. S1: [file JCMM-27-2045-s001.tif]
